# Thyroid nodule as a first manifestation of Hodgkin lymphoma–report of two cases and literature review

**DOI:** 10.1186/1746-1596-8-116

**Published:** 2013-07-15

**Authors:** Ewelina Szczepanek-Parulska, Malgorzata Szkudlarek, Przemyslaw Majewski, Jan Breborowicz, Marek Ruchala

**Affiliations:** 1 Department of Endocrinology, Metabolism and Internal Medicine, Poznan University of Medical Sciences, Poznan, Poland; 2 Department of Clinical Pathology, Poznan University of Medical Sciences, Poznan, Poland; 3 Department of Oncology, Poznan University of Medical Sciences, Poznan, Poland

**Keywords:** Thyroid nodule, Hodgkin lymphoma, Fine-needle aspiration biopsy (FNAB), Reed-Sternberg cells

## Abstract

**Abstract:**

Lymphomas account for less than 5% of thyroid malignant lesions. Vast majority of them are B-cell non-Hodgkin lymphomas (NHL), while Hodgkin lymphoma (HL) is extremely rare. Here we present two cases of HL, at baseline manifesting as a thyroid lesion. First patient, 29-year-old pregnant female, initially suspected for metastatic medullary thyroid cancer, was eventually diagnosed with mixed cellularity type of thyroid HL. Second patient, 22-year-old woman with suspicion of advanced thyroid cancer, was in the end diagnosed with an extra-lymphatic classical HL of the thyroid. In both cases, despite repeated fine-needle aspiration biopsy, cytological examination gave inconclusive or misleading results. On histopathological examination, thyroid tumor cells were positive for CD15 and CD30 antigen, which is typical for Reed-Sternberg cells. In the report authors also discuss difficulties in management as well as potential importance of novel methods such as FISH, PCR and other molecular techniques in diagnostics of thyroid lymphomas.

**Virtual slides:**

The virtual slide(s) for this article can be found here: http://www.diagnosticpathology.diagnomx.eu/vs/2896947559559648

## Background

Hodgkin lymphomas (HL) is a biologically heterogeneous group of neoplasms. The incidence of HL in European Union is estimated at about 2.2-2.7/100 000 cases per year, constituting 11.7% of all lymphomas diagnosed in 2006 [1]. The majority of cases is in low-stage disease and presents the nodular sclerosis subtype.

An increase in incidence of extra-nodal lymphomas has been observed over the past two decades [2]. Extra-nodal origin is more common in NHL and may reach 33% [3]. On the other hand, HL mainly arises in lymph nodes of the neck and mediastinum, while only approximately 5% develop in extra-nodal sites, including tonsils, nasopharynx, parotid glands, thyroid, parathyroid or nasal antrum, with or without concomitant nodal involvement [4].

The head and neck is a third place of extra-nodal localization of the lymphomas [5]. Lymphomas account for less than 5% of malignant lesions diagnosed in the thyroid identified in about two cases per million [6,7]. Vast majority of them are B-cell non-Hodgkin lymphomas (NHL), developing in the course of autoimmune thyroiditis, while HL primarily localized in the thyroid, is a very rare finding [8,9].

Extra-nodal lymphomas (ENL) represent different pathologic, imaging, and clinical features as well as dissimilar prognosis from nodal lymphomas, and should therefore be distinguished [10]. Extra-nodal involvement is much less common in HL compared with NHL. ENL more often spreads by continuity from contiguous nodal disease, while haematogenous spread is rare, occurring in 5%-10% of patients [11].

Hodgkin lymphoma of the thyroid is uncommon and accounts for 0.6-5% of all thyroid malignancies and 2-7% of all ENL [12-15]. In vast majority primary thyroid lymphomas are of B-cell origin. Only 1-2% of thyroid lymphomas derive from T-cell lymphocyte [12,16].

Factors leading to the development of thyroid lymphoma are not fully understood. Some authors attempt to relate a significant prevalence of women with thyroid HL to autoimmune thyroiditis [16]. It is considered, that thyroid lymphoma develops from lymphocytic tissue present in the gland only in case of autoimmune thyroid disease, while normal thyroid gland is devoid of native lymphoid tissue [17]. Hashimoto thyroiditis is detected in 27% to 100% of thyroid lymphoma cases [18]. On the other hand, less than 1% of patients suffering from Hashimoto disease come down with lymphoma [19,20]. It is estimated, that a period of 20 to 30 years is needed for lymphoma to develop in case of thyroiditis [17]. In the largest study of patients with HL by Wang et al., 7 out of 21 subjects presented Hashimoto thyroiditis [8].

Among factors, which may play role in the development of thyroid lymphoma, the prolonged antigen stimulation as well as aberrant somatic hypermutation are mentioned [21-24]. Ebstein-Barr virus (EBV) infection is also mentioned as etiological factor, since 20-100% of systemic HL is thought to be associated with EBV infection; more likely in mixed cell and lymphocyte depletion types of HL. Moreover, HHV-6 can be detected in 48% of Reed-Sternberg (R-S) cells of nodular sclerosis HL and is considered as another factor potentially promoting development of this type of HL [25].

It remains a matter of debate whether thyroid gland in these cases is a primary site of HL origin or a secondary organ involved in the disease [8]. Organ specific HL without concomitant lymph nodes involvement is extremely rare [26]. In our paper we aim to focus on subjects, in whom the diagnosis of HL was made in the course of evaluation of a thyroid lesion and later on involvement of other sites was detected.

Here we present two cases of Hodgkin lymphoma, initially manifesting as thyroid lesions.

## Case report

### Patient 1

**Figure 1:**
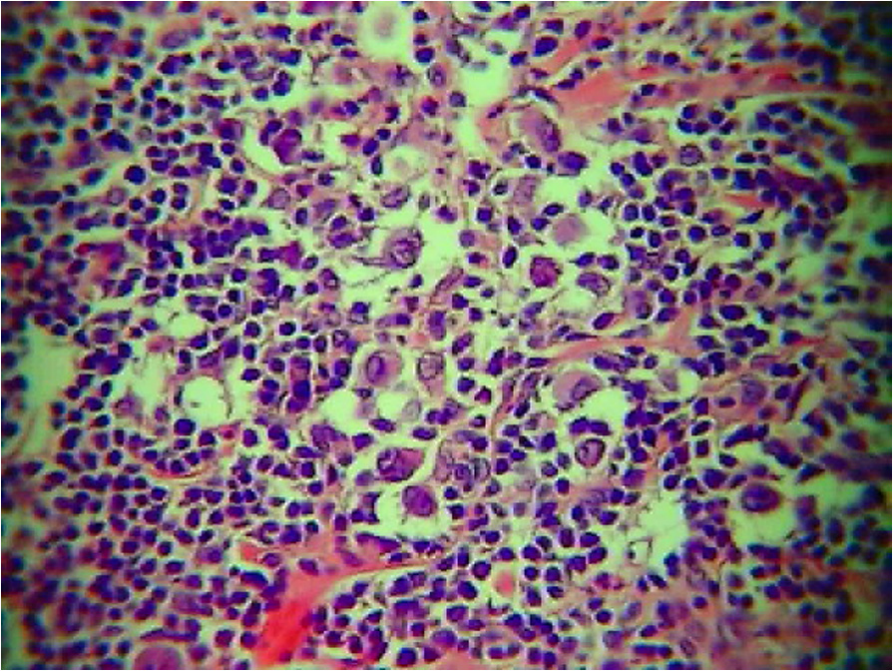
Histopathological examination of the material obtained during thyroidectomy - Hodgkin lymphoma - mixed cellularity (MC) type in Patient 1 (H&E, magnification × 10).

**Figure 2:**
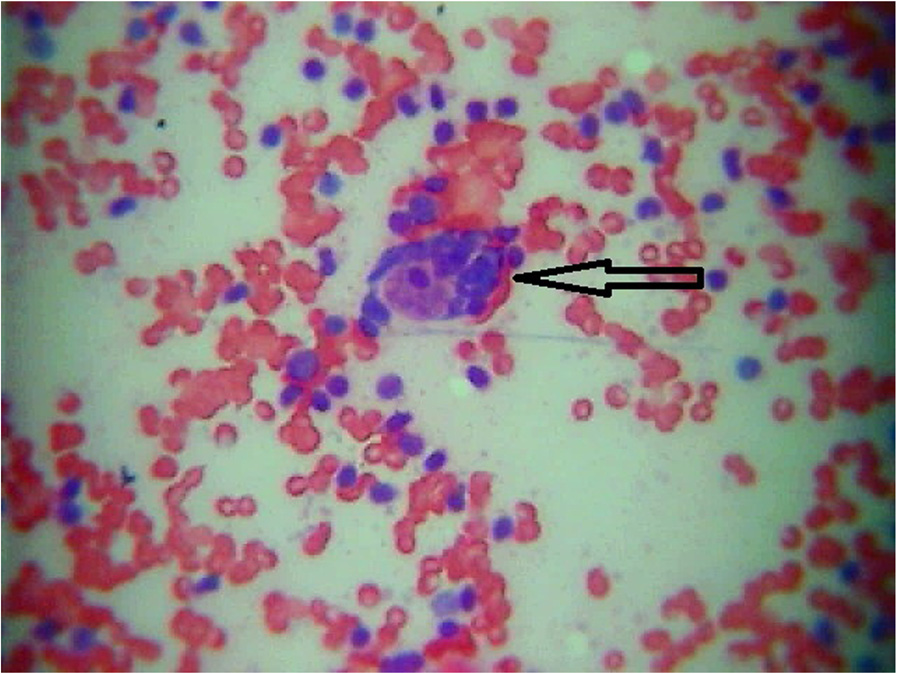
**Retrospective verification of cytological specimen obtained preoperatively during fine-needle aspiration biopsy of the thyroid in the Patient 1.** An arrow indicates Reed-Sternberg cell, presence of which might provide preoperative diagnosis of Hodgkin lymphoma (H&E, magnification × 400).

In April 2002, 29-year-old pregnant woman was referred for ultrasound examination of the neck due to considerably enlarged cervical lymph nodes detected on palpation. On sonography the packages of lymph nodes with coinciding hypoechogenic ill-defined thyroid lesion were demonstrated, what raised suspicion of advanced thyroid cancer. Patient underwent fine-needle aspiration biopsy (FNAB) of both thyroid lesion and lymph nodes. The result of cytological examination was consistent with suspicion of medullary thyroid cancer. At the time of diagnosis, the patient was 29 weeks pregnant with her first baby. Due to suspicion of metastatic medullary thyroid carcinoma, decision on surgical treatment was made, despite the patient was in the third trimester. The total thyroidectomy with lymphadenectomy was performed, with coordinated care of an anesthesiologist and a gynecologist, constantly monitoring the welfare of both mother and fetus. Perioperative period was uneventful. Post-operative immunohistochemical and histopathological examinations revealed, that previously observed immunostaining for calcitonin was falsely positive in lacunar cells and lymphocytes. Thyroid tumor cells expressed antigen CD15 and CD30, which is typical for R-S cells, being a marker of HL. Thus, final diagnosis of mixed cellularity (MC) type HL of the thyroid was established (Figure 1). Only retrospective verification of the specimen obtained during preoperative FNAB, revealed the presence of R-S cell (Figure 2). Consulting hematologist decided to postpone chemotherapy for the time after delivery. In the 36th week of pregnancy, the symptoms of preterm labor occurred and the patient delivered a healthy boy by caesarean section. Three weeks later the patient was admitted to the department of hematology, where thorough clinical evaluation, bone marrow biopsy and imaging studies were performed to establish the advancement of the disease. The patient presented involvement of cervical and thoracic lymph nodes, splenomegaly and clinical symptoms (fever >38°C and sweating), hence IVB stage of HL according to Ann Arbor classification was diagnosed. She received 8 courses of ABVD (Adriamycin, Bleomycin, Vinblastine, Dacarbazine) chemotherapy and reached remission. During 10-year follow-up period, no relapse of lymphoma was detected. Four years following the therapy, the patient got pregnant and gave birth to healthy female twins. She is still followed up in an endocrine outpatient clinic and her hormonal balance is maintained with 100 μg of L-thyroxin.

### Patient 2

**Figure 3:**
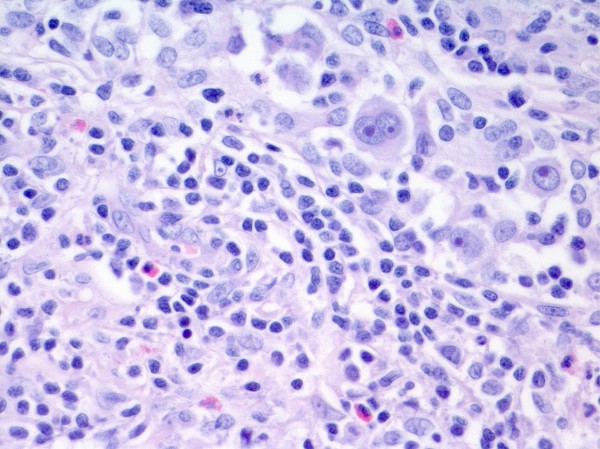
Histopathological examination of the material obtained during thyroidectomy - Hodgkin lymphoma - nodular sclerosis (NS) type in Patient 2 (H&E, magnification × 10).

**Figure 4:**
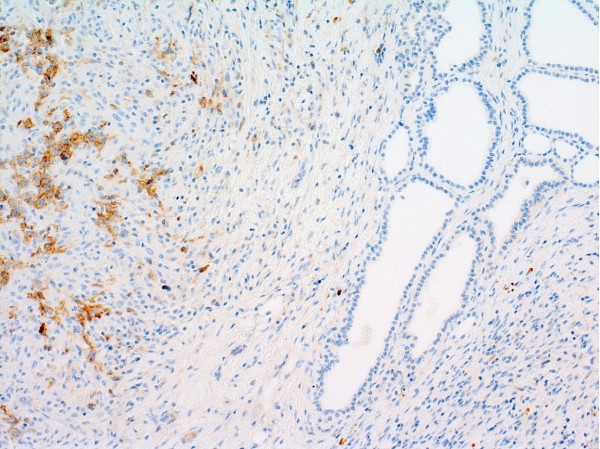
Reed-Sternberg cells positive for CD 30 in Patient 2 (H&E, magnification × 10).

23-year-old woman came to a laryngologist with painless bilateral enlargement of neck lymph nodes and hoarseness. Despite prescribed antibiotic therapy, symptoms persisted, thus ultrasound examination of the neck was performed, which demonstrated thyroid lesion with concomitant involvement of lymph nodes. Subsequently the patient was referred to the endocrinology outpatient clinic with suspicion of advanced thyroid cancer in September 2008. On thyroid ultrasound examination, a large (32x30 mm), hypoechogenic lesion, localized at the border between left thyroid lobe and isthmus, was visualized. Unilaterally, package of enlarged, round and hypoechogenic lymph nodes of size 20×12 mm was found, suggesting metastatic lesions. The trachea was displaced to the right side. On thyroid scintiscan, a large cold nodule was found and its localization corresponded to the lesion revealed during ultrasonography. The chest X-ray disclosed the enlargement of the upper mediastinum (to 70 mm). There was no past history of thyroid or hematologic disease. Her family history was non-contributory. At the time of diagnosis the patient was euthyroid and thyroid autoantibodies were negative. Other laboratory tests revealed accelerated ESR and increased concentration of white blood cells with depletion of lymphocytes and eosinophils. Thyroid and lymph nodes FNAB was performed three times. The cytological examination of initial two specimens gave non-diagnostic result due to too small amount of cells obtained in the specimens. Eventually, the third biopsy allowed to detect suspected cells of undetermined origin. The clinical picture, together with results of imaging studies and cytological examination, prompted us to refer the patient for immediate total thyroidectomy with lymphadenectomy. Immunohistochemical studies were performed and showed the tumor cells expressing CD30+, CD15+, Ki67+. Tumor cells were negative for D3-, CD20-, CKAE1/3, EBV-LMP1. Thus a final diagnosis of extra-lymphatic classical HL (nodular sclerosis subtype) of the thyroid was made (Figures 3 and 4).

The patient was subsequently referred to the department of hematology. On the base of clinical picture, imaging examinations and bone marrow assessment, she was classified for stage IIE of disease in Ann Arbor classification. The patient was subjected to combined chemotherapy (12 cycles of ABVD) and radiotherapy, starting from December 2008. The following imaging studies, including chest X-ray and positron emission tomography were performed to confirm remission status and did not reveal any pathological changes. The patients observation period has now reached four years and is uneventful. She continues to be followed-up in an endocrinology outpatient clinic and remains both clinically and biochemically euthyroid on substitutive 125 μg dose of L-thyroxin.

## Discussion

Neoplasms diagnosed in the thyroid gland are usually primary thyroid cancers, while lymphomas account for less than 5% of malignant lesions diagnosed in the thyroid. Vast majority of them are B-cell non-Hodgkin lymphomas (NHL), developing in the course of autoimmune thyroiditis, while Hodgkin lymphoma (HL), primarily localized in the thyroid, is a very rare finding [12-14]. However, in differential diagnosis also rarely occurring tumours of this localization need to be involved [27-31]. The thyroid gland constitutes an uncommon site for metastatic changes from various primary sites. Thyroid metastases are encountered in 2% to 24% of the patients with malignant neoplasm [28]. Differential diagnosis of thyroid lesions should also comprise other uncommon primary thyroid tumors. Leiomyosarcomas of the thyroid account for 6% of the all head and neck tumors, with 18 cases described so far in the world literature [29]. Primary squamous cell carcinoma (SCC) of the thyroid is also an extremely rare entity, observed in less than 1% of all thyroid malignancies [30]. Vascular lesions include benign tumors such as hemangiomas and malignant ones including angiosarcomas or undifferentiated angiosarcomatoid carcinomas [31].

Our extensive literature search revealed thirty seven cases of HL located in the thyroid gland reported to date. In patients with thyroid HL women predominate and constitute about 75-80% of reported patients [4,32], while systemic HL has an equal male to female ratio. In the paper describing the largest series of subjects with thyroid HL by Wang et al., an average age of a patient with HL of the thyroid gland was 42 (with range from 18 to 64) and was lower than for NHL (7th decade) [8]. About two thirds of patients suffering from thyroid HL were younger than 45 years old [33]. It is consistent with what we learned from our patients, who were diagnosed even below the age of thirty.

As we mentioned above, autoimmune thyroiditis is one of the factors linked with development of lymphomas. Our two patients with thyroid HL presented neither laboratory nor cytological features of thyroid inflammation. Thyroid HL in over 80% of patients manifests as a rapidly enlarging unilateral or bilateral neck mass. Other clinical symptoms include: hoarseness (35%), dyspnea (65%) and dysphagia (53%) [28]. HL of the thyroid may present as diffuse involvement of the gland or a focal lesion [29]. Both our patients presented enlargement of neck lymph nodes and the second one also has been complaining about hoarseness. Similarly to our patients, on physical examination in most cases thyroid tumor tends to be firm to hard upon palpation [30]. Vast majority of formerly reported patients with HL presented concomitant neck lymph nodes involvement at the time of diagnosis, but the presence of B-symptoms, including inter alia fever and sweating occurring in one of our patients, at the same time is relatively rare (33%) [16]. B-symptoms may develop in the course of lymphoma and are usually associated with systemic involvement and constitute a marker of negative prognosis. These include temperature >38°C (>100.4°F) for three consecutive days, weight loss exceeding 10% of body weight in 6 months and drenching night sweats.

Patients with thyroid HL are mostly euthyroid or less often hypothyroid (30-40%) at the time of diagnosis [34]. Both our patients presented normal thyroid function. On scintiscan HL localized in the thyroid presents as a cold nodule, while ultrasound examination reveals diffused or focal thyroid enlargement, mimicking thyroiditis or primary thyroid lesion [8]. In case of the first patient, scintiscan was contraindicated because of pregnancy. A large cold nodule was found on thyroid scintiscan of the second patient. The involved lymph nodes are homogeneous, variable in size and might display necrosis and calcifications [4]. On ultrasound examination, enlarged lymph nodes were found in both described cases. However, despite increased size and formation of packages, no other suspected features of lymph nodes were demonstrated.

The difficulty in diagnosis poses the fact, that if the personal history is negative for hematologic diseases, the thyroid lymphoma is rarely suspected. Diagnosis of lymphoma should be considered when dealing with rapidly enlarging goitre. The FNAB constitutes the initial examination used for evaluation of thyroid lesion. Its role is limited but the procedure is necessary for both immunocytochemical studies and flow cytometry. Primary thyroid HL should be suspected when the specimen consists of lymphocytes, but carcinoma cannot be excluded. The reports on the efficacy of FNAB in diagnostics of thyroid lymphomas provide diverse results. According to different studies, 50-80% of cases of thyroid lymphomas is revealed on the basis of FNAB results [7,13,35-38]. However, Gupta et al. reported that in nine out of ten cases, a diagnosis of NHL was made on the base of FNAB [13]. Only in one case, in a patient presenting diffuse swelling, cytology demonstrated a polymorphic infiltrate consistent with reactive hyperplasia and the diagnosis of lymphoma was missed [13]. In the study by Dedecjus et al., the proper diagnosis of thyroid lymphoma was made in less than 50% of cases in US-guided FNAB, while in other study by Seningen et al., the FNAB characterized with very high specificity and positive predictive value at the level of 99.6% and 88.9%, respectively [3,39]. In case of a primary thyroid NHL, diagnosis of large cell type lymphoma is simple on FNAB due to build of large cells, lack of cellular cohesion and lymphoglandular bodies in the background [13]. On the contrary, diagnosis of MALT- lymphomas basing on cytology is difficult due to heterogeneous appearance of the neoplastic infiltrate [40].

Differentiation of primary thyroid lymphoma from thyroid cancer and Hashimoto thyroiditis may produce a diagnostic dilemma. Low accuracy of FNAB comes out of hypocellular samples, small population of R-S cells, marked fibrosis and sclerosis, resemblance between the R-S cells and inflammatory cells and, finally, very rare incidence of thyroid HL [8]. Immunocytochemistry should be used to confirm the suspicion of lymphoma [41]. Small biopsy probes may lead to diagnosis of thyroiditis instead of lymphoma, due to presence of neutrophils, abscess or necrosis [42]. It remains a challenge to differentiate between benign and malignant lymphoid infiltrate [19]. FNAB constitutes a helpful initial diagnostic device to establish the preliminary diagnosis, however is not sufficient for the final one, because the evaluation of lymph node architecture is extremely important [43,44]. What is more, in HL the tumour cells represent the minority of the cellular population, where normal reactive lymphocytes, eosinophils, and histiocytes predominate. Hence, cytological specimen obtained during FNAB may not contain the R-S cells, indispensable for adequate diagnosis [44]. Lymphomas may differ from Hashimoto thyroiditis with the abundance of lymphoid tissue and a high proportion of intermediate centrocyte-like cells, especially in low-grade NHL. A sampling error and coexistence of Hashimoto thyroiditis and lymphoma might cause false-negative results [40].

The diagnostic accuracy of FNAB of lymph node is evaluated from 30 to 92%. The main limitations are fibrosis and abundant benign surroundings [42,45]. It is reported in the literature that a single-tube flow cytometry can be used in screening of classic HL in tissue body sections or FNAB specimens with 88% sensitivity and even 100% specificity [42,46]. Other corroborative methods include tissue biopsy and surgery.

Contemporary development in diagnostics allow to increase the diagnostic accuracy of cytological examination in lymphomas. Immunocytochemistry allows for confirmation of the lymphoid origin of the cells and their B or T-lineage [13]. Flow cytometry is a well-adapted method to establish the immunophenotype in FNAB specimens [19,47]. In case of lymph nodes, flow cytometry characterizes with high sensitivity and specify, ranging from 94 to 100% [47-50]. However, in flow cytometry-based diagnosis of the thyroid gland lesions, elevated κ:λ ratios have been observed in many case of thyroiditis, what can produce a diagnostic dilemma [51].

Ochs et. al stress that genetic testing, including polymerase chain reaction (PCR) and florescence in situ hybridization (FISH) is feasible and useful tool in diagnosis of thyroid lymphomas [19]. Identification of genetic abnormalities can be critical to the diagnosis of lymphoma, especially this of unusual site of origin like lymphomas of thyroid gland [19]. Endocrinologists frequently have to make a decision solely on the basis of FNAB hence the use of molecular testing in limited material obtained during FNAB is very helpful to establish diagnosis prior to invasive procedures [19]. PCR-based methods constitute important diagnostic method in cases when flow cytometry does not allow to reveal clonality of the cells. Clonality testing can be applied for indicating the relationship between sites in multifocal disease [19]. R-S cells account for only 0.1-1% of the cells in material obtained during FNAB [52]. Molecular techniques targeting these cells provide some information on cytogenetic aberrations in HL [52]. However, immunoglobulin heavy chain genes should be interpreted with attention due to frequency of monoclonal rearrangement in thyroiditis [53].

Genomic gains in chromosom arm 2p, including REL gene and gains in 9p are present in 30 to 50% of classic HL [54-57]. Chromosomal breakpoints affecting immunoglobulin loci are recurrent in B-cell lymphomas, but also classical HL. In about 17% of R-S cells, Martin-Subero et al. observed breakpoints in IGH, IGL or IGK locus [52,57].

Other molecular cytogenic technics useful in diagnostic process of HL apart from FISH and PCR include comparative genomic hybridization (CGH) from microdissected R-S cells, fluorescence immunophenotyping and interphase cytogenetics (FICTION) as a tool for the investigation of neoplasms [52].

In case of our two patients FNAB failed to bring the preoperative diagnosis of HL. Unfortunately, none of the above described advanced techniques supporting cytological examination were at the moment of evaluation of the described patients a part of routine diagnosis at our department. In Patient 1, non-specific staining for calcitonin resulted in misdiagnosing medullary thyroid cancer, and only during the retrospective re-evaluation of the cytological material obtained during FNAB, R-S cell was identified. Incidence of calcitonin-containing cells in thyroid lymphoma and in Hashimoto thyroiditis was already described by Baschieri et al. [58]. C cell hyperplasia is present frequently in lymphomas and shows positivity for calcitonin. Hyperplastic C cells are not observed in Hashimoto thyroiditis. Hence, an increase in the C cell number might be a marker of thyroid lymphoma [58].

In Patient 2, the repeated FNAB did not allow to obtain the number of cells high enough to establish diagnosis. Hence, without clinician’s suggestion and experience of pathologists, possibility of HL diagnosis in the thyroid gland is limited.

Final pathological diagnosis should be made from surgical specimen or excisional lymph node biopsy and should be based on WHO classification [25,59]. One of the basis of histopathological diagnosis of HL is the identification of the presence of R-S cells. These are large mutated cells, derived from B lymphocytes, presenting with an amphophylic cytoplasm and multiple or a bilobed nucleus, eosinophilic inclusion-mimicking nucleoli, devoid of typical B-cell markers such as CD20 and CD79a. Their characteristic immunophenotype is of crucial importance in diagnosis of HL. A CD 30 antigen is typical for R-S cells, while CD 15, an antigen normally expressed in monocytes and granulocytes, but not in lymphocytes, is present at R-S cells in 75-85%, independent of the type of HL [60,61].

Classical HL, which constitutes about 95% of all HL cases, can be divided into nodular sclerosis, mixed cellularity, lymphocyte-rich and lymphocyte depleted subtypes [25]. Staging is estimated according to the Ann Arbor system and it determines prognosis and validity of radiotherapy [59]. Most patients present stage I or II of the disease and the nodular sclerosis subtype [8]. There were only two cases of mixed cellular subtype of HL involving the thyroid reported so far in the literature and to our knowledge, one of our patients would be a third case [8,62].

The literature search reveals that surgical removal of the thyroid affected with HL does not influence the prognosis [22,44]. HL responds quickly to chemotherapy and radiotherapy, while surgical risk is considered unnecessary [22,32]. However, excision of the lesion can be of importance in cases with acute airway obstruction [34]. Preoperative diagnosis is not always established, hence, due to similar clinical picture of hypoechogenic lesion of the thyroid, presenting as cold nodule in the scintiscan, the patient is referred for thyroid surgery with suspicion of primary thyroid cancer, as it was in case of both our patients. The nodal involvement additionally suggests metastatic disease. Hence, if the presurgical diagnosis of HL is reached, the operation is not recommended. However, in both our cases, cytological examinations was not conclusive, hence surgery and histopathological examination was the only way to determine the diagnosis.

With appropriate therapy, primary thyroid HL is associated with favourable prognosis. Nowadays, chemotherapy (ABVD) and 30 Gy radiotherapy is the treatment of choice in primary thyroid lymphoma [25,63]. The final outcome depends on the histological type and stage of the disease at presentation. The survival is significantly greater for HL than for NHL [16]. It was determined, that both high value of ESR (over 50 mm/h without systemic symptoms) and extra-nodal involvement independently constitute important pretreatment prognostic factors in subjects with HL [2,64]. One of our patients presented markers of unfavourable prognosis such as ESR 71 mm/h and both had extra-nodal localization of the lymphoma. However, at the moment of publication, both patients were in remission for ten and four years, respectively.

## Conclusions

To conclude, despite its rarity, Hodgkin lymphoma ought to be considered in differential diagnosis of thyroid lesions. An appropriate preoperative diagnosis prevents from unnecessary total thyroidectomy. Hodgkin lymphoma that initially presents as a thyroid mass, treated with combined modality therapy has favourable outcome.

## Consent

Written informed consent was obtained from the patient for publication of this Case Report and any accompanying images. A copy of the written consent is available for review by the Editor-in-Chief of this journal.

## Competing interests

The authors declare that they have no competing interests.

## Authors’ contributions

ESP preparated manuscipt, performed the literature review. MS preparated manuscipt, performed the literature review. PM performed histopathological examinations and documentation, acquired photomicrographs. JB gave the final histopathological diagnosis and prepared the documentation. MR participated in patient management and corrected the manuscript and approved of the final version of the manuscript. All authors read and approved the final manuscript.
